# Supplementary material for: Seed Size-Number Trade-Off Exists in Graminoids but Not in Forbs or Legumes: A Study from 11 Common Species in Alpine Steppe Communities
Source: Plants (Basel). 2025 Sep 2;14(17):2730. doi: 10.3390/plants14172730 (PMC12430029; doi:10.3390/plants14172730)
Supplement: Supplementary file 1 [file plants-14-02730-s001.zip › plants-3801640-supplementary.pdf]

Supplementary Materials:

Table S1. The results of linear regression and linear regression based on PICs (phylogenetically independent contrasts) between mean value of species' seed size and seed number in community (Bold indicate statistically significant differences ($p<0.05$)).

Year	Linear Regression			Linear Regression Based on PICs		
	Slope	R^2	*p*	Slope	R^2	*p*
2018	−0.88	0.47	**0.02**	−0.67	0.34	**0.04**
2019	−0.74	0.60	**0.02**	−0.64	0.45	0.06

Table S2. The PCA loadings for each seed trait.

Seed Trait	PC1 Loadings	PC2 Loadings
seed number	0.82307	−0.07462
seed size	−0.79715	−0.35285
seed length	−0.24225	−0.73452
seed width	−0.83016	−0.33494
seed height	−0.86452	0.44045
seed C content	0.07778	0.86364
seed N content	0.37279	−0.26882
seed P content	0.77094	−0.33989

Table S3. The raw data of eight seed traits for article analysis (C: seed carbon content, N: seed nitrogen content, P: seed phosphorus content).

Species	Seed Number	Seed Size (mg)	Length (mm)	Width (mm)	Height (mm)	C (%)	N (%)	P (%)
Oxytropis_glabra	22.63	1.13	1.99	1.56	0.77	54.55	6.45	0.50
Astragalus_alpinus	54.1	1.13	1.829	1.49	0.66	46.53	6.86	0.67
Stipa_purpurea	22	2.64	5.459	1.08	1.02	54.87	2.78	0.42
Poa_crymophila	272.64	0.2	1.16	0.69	0.22	52.09	2.70	0.47
Festuca_ovina	1772	0.28	2.92	0.72	0.38	53.00	3.96	0.50
Koeleria_cristata	538.55	0.13	3.40	0.78	0.06	47.44	4.10	0.99
Agropyron_cristatum	34	1.05	1.41	0.59	0.54	60.19	5.79	0.81
Potentilla_bifurca	35.63	0.34	1.44	1.15	0.92	54.68	1.42	0.34
Leontopodium_ leontopodioides	308	0.05	1.31	0.56	0.33	61.88	6.60	0.93
Potentilla_multifida	91.09	0.13	1.61	1.14	0.76	54.74	2.57	0.39
Potentilla_fragarioides	653.9	0.24	1.32	0.94	0.60	55.74	5.26	0.52

Table S4. The raw data of reproductive biomass, seed number, and seed mass for article analysis.

Species	Reproductive Biomass (g)	Seed Number	Seed Size (mg)	Functional Group
Oxytrois_glabra	0.1307	23	1.382609	Legum
Oxytrois_glabra	0.3706	63	1.350794	Legum
Oxytrois_glabra	0.0564	12	0.816667	Legum
Oxytrois_glabra	0.1233	16	1.0375	Legum
Oxytrois_glabra	0.1128	13	0.984615	Legum

Oxytrois_glabra	0.0799	16	1.46875	Legum
Oxytrois_glabra	0.0718	9	1.1	Legum
Oxytrois_glabra	0.0425	12	1.125	Legum
Oxytrois_glabra	0.0565	5	0.88	Legum
Oxytrois_glabra	0.1422	7	0.814286	Legum
Oxytrois_glabra	0.7849	73	1.469863	Legum
Astragalus_multicaulis	0.0382	28	1.053571	Legum
Astragalus_multicaulis	0.0767	14	1.335714	Legum
Astragalus_multicaulis	0.5019	94	1.380851	Legum
Astragalus_multicaulis	0.1016	35	1.011429	Legum
Astragalus_multicaulis	0.215	45	1.286667	Legum
Astragalus_multicaulis	0.1508	46	1.295652	Legum
Astragalus_multicaulis	0.079	17	0.876471	Legum
Astragalus_multicaulis	0.1719	64	1.176563	Legum
Astragalus_multicaulis	0.235	114	0.734211	Legum
Astragalus_multicaulis	0.215	84	1.153571	Legum
Potentilla_multifida	0.0233	93	0.097849	Forb
Potentilla_multifida	0.0186	32	0.09375	Forb
Potentilla_multifida	0.0192	50	0.126	Forb
Potentilla_multifida	0.0465	112	0.129464	Forb
Potentilla_multifida	0.0124	9	0.188889	Forb
Potentilla_multifida	0.0329	142	0.116197	Forb
Potentilla_multifida	0.0213	48	0.133333	Forb
Potentilla_multifida	0.0748	300	0.125667	Forb
Potentilla_multifida	0.039	113	0.199115	Forb
Potentilla_multifida	0.0115	100	0.131	Forb
Potentilla_multifida	0.014	3	0.1	Forb
Poa_crymophila	0.0397	74	0.17973	Graminoid
Poa_crymophila	0.0754	115	0.231304	Graminoid
Poa_crymophila	0.2717	1428	0.173109	Graminoid
Poa_crymophila	0.0673	177	0.158757	Graminoid
Poa_crymophila	0.1137	346	0.154046	Graminoid
Poa_crymophila	0.0858	201	0.235323	Graminoid
Poa_crymophila	0.0349	83	0.206024	Graminoid
Poa_crymophila	0.0293	79	0.187342	Graminoid
Poa_crymophila	0.039	109	0.202752	Graminoid
Poa_crymophila	0.028	43	0.251163	Graminoid
Poa_crymophila	0.226	344	0.22064	Graminoid
Festuca_ovina	0.2495	3234	0.265121	Graminoid
Festuca_ovina	0.0572	522	0.332184	Graminoid
Festuca_ovina	0.0422	636	0.227358	Graminoid
Festuca_ovina	0.0928	1272	0.286321	Graminoid
Festuca_ovina	0.092	1956	0.238957	Graminoid
Festuca_ovina	0.2663	2610	0.345057	Graminoid
Festuca_ovina	0.3495	3858	0.316952	Graminoid
Festuca_ovina	0.0216	342	0.236842	Graminoid
Festuca_ovina	0.2798	1518	0.253755	Graminoid
Potentilla_fragarioides	0.4291	894	0.237136	Forb
Potentilla_fragarioides	0.4694	976	0.25584	Forb
Potentilla_fragarioides	0.2449	518	0.251544	Forb
Potentilla_fragarioides	0.2815	392	0.316327	Forb

Potentilla_fragarioides	0.1571	423	0.201182	Forb
Potentilla_fragarioides	0.1629	322	0.229503	Forb
Potentilla_fragarioides	0.219	258	0.288372	Forb
Potentilla_fragarioides	0.33	777	0.237066	Forb
Potentilla_fragarioides	0.1664	367	0.233515	Forb
Potentilla_fragarioides	0.4743	1089	0.196143	Forb
Potentilla_fragarioides	0.3533	755	0.260795	Forb
Potentilla_fragarioides	0.5403	1076	0.216357	Forb
Koeleria_cristata	0.0264	1024	0.100391	Graminoid
Koeleria_cristata	0.0829	1424	0.133708	Graminoid
Koeleria_cristata	0.0356	832	0.145673	Graminoid
Koeleria_cristata	0.0472	584	0.128767	Graminoid
Koeleria_cristata	0.0177	276	0.162319	Graminoid
Koeleria_cristata	0.0223	288	0.126389	Graminoid
Koeleria_cristata	0.0282	252	0.11746	Graminoid
Koeleria_cristata	0.0516	384	0.146875	Graminoid
Koeleria_cristata	0.0191	140	0.165714	Graminoid
Koeleria_cristata	0.0627	524	0.135878	Graminoid
Koeleria_cristata	0.0364	196	0.112245	Graminoid
Stipa_purpurea	0.1452	69	2.886957	Graminoid
Stipa_purpurea	0.0528	15	2.88	Graminoid
Stipa_purpurea	0.3012	171	2.722807	Graminoid
Stipa_purpurea	0.092	36	2.666667	Graminoid
Stipa_purpurea	0.1299	45	2.62	Graminoid
Stipa_purpurea	0.1225	60	2.765	Graminoid
Stipa_purpurea	0.0412	24	2.5875	Graminoid
Stipa_purpurea	0.2816	216	2.1	Graminoid
Stipa_purpurea	0.1076	99	2.390909	Graminoid
Stipa_purpurea	0.1947	99	2.769697	Graminoid
Agropyron_cristatum	0.0831	45	1.048889	Graminoid
Agropyron_cristatum	0.1029	55	1.378182	Graminoid
Agropyron_cristatum	0.0646	47	1.002128	Graminoid
Agropyron_cristatum	0.0348	25	1.112	Graminoid
Agropyron_cristatum	0.0243	8	0.825	Graminoid
Agropyron_cristatum	0.0586	46	0.865217	Graminoid
Agropyron_cristatum	0.0564	6	1.2	Graminoid
Agropyron_cristatum	0.0358	25	1.08	Graminoid
Agropyron_cristatum	0.0465	20	1.04	Graminoid
Agropyron_cristatum	0.0909	63	0.939683	Graminoid
Leontopodium_leontopodioides	0.2242	13	0.053846	Forb
Leontopodium_leontopodioides	0.0638	174	0.035057	Forb
Leontopodium_leontopodioides	0.8468	71	0.076056	Forb
Leontopodium_leontopodioides	0.0672	6	0.066667	Forb
Leontopodium_leontopodioides	0.4459	59	0.052542	Forb
Leontopodium_leontopodioides	0.1065	86	0.053488	Forb

Leontopodium_leontopodioides	0.3173	53	0.060377	Forb
Leontopodium_leontopodioides	0.1509	109	0.055046	Forb
Leontopodium_leontopodioides	0.0871	6	0.016667	Forb
Potentilla_bifurca	0.0341	63	0.304762	Forb
Potentilla_bifurca	0.027	49	0.312245	Forb
Potentilla_bifurca	0.0129	13	0.476923	Forb
Potentilla_bifurca	0.0192	41	0.302439	Forb
Potentilla_bifurca	0.0197	40	0.31	Forb
Potentilla_bifurca	0.0215	20	0.32	Forb
Potentilla_bifurca	0.0168	23	0.413043	Forb
Potentilla_bifurca	0.0178	36	0.305556	Forb

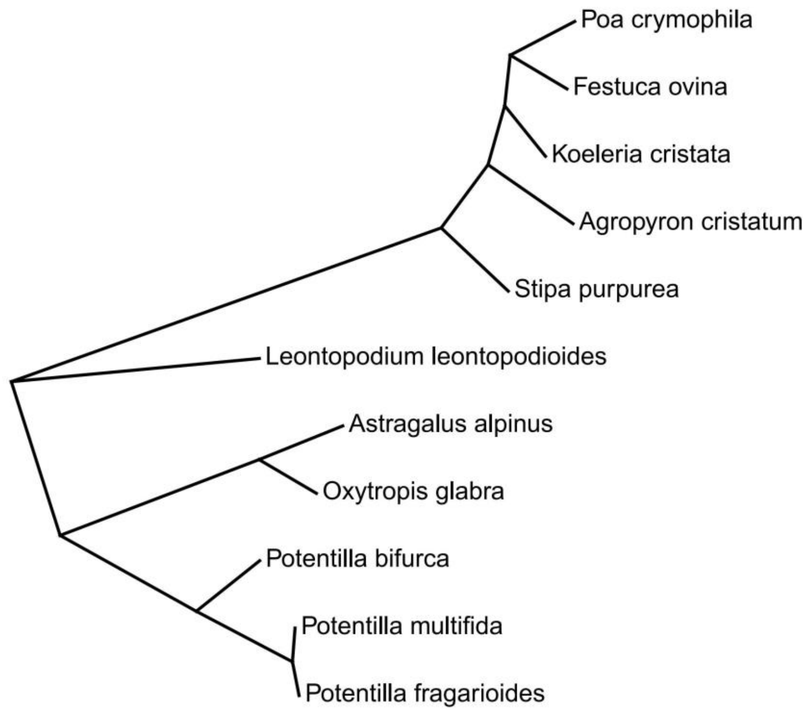

Figure S1. The phylogenetic tree based on nuclear ITS sequences of species.

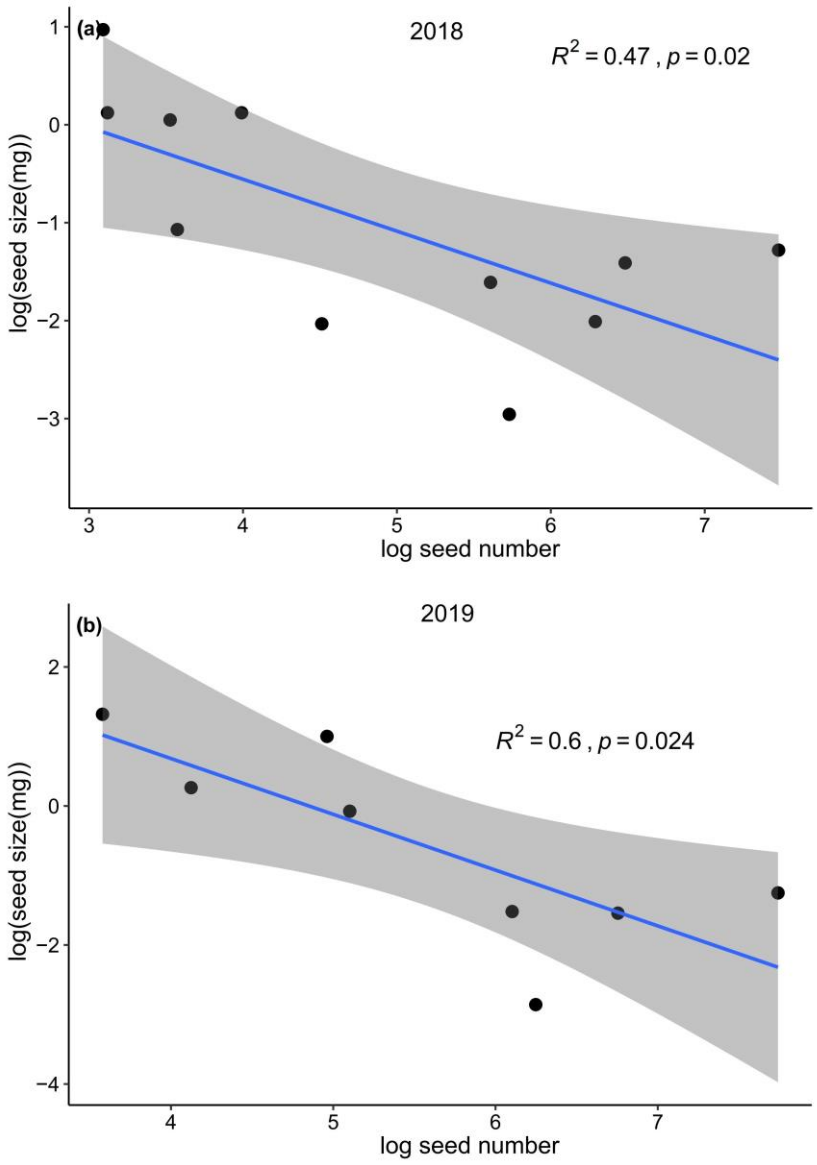

Figure S2. The relationship between seed size and seed number of different species in community in 2018 (a) and 2019 (b). Each point represents a species' mean value.

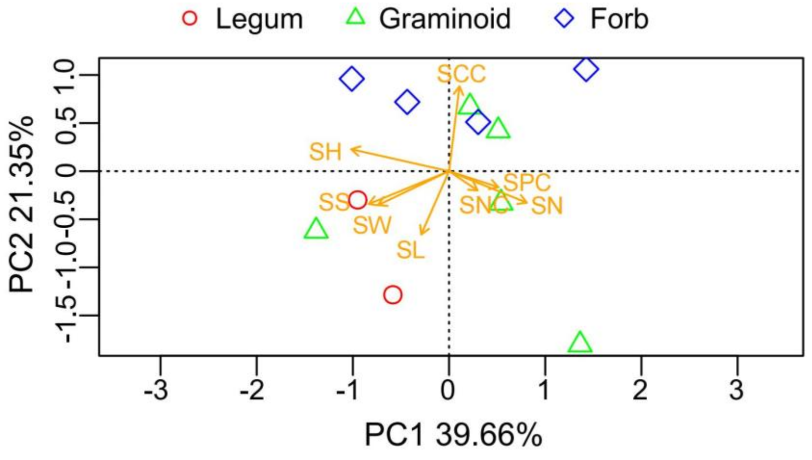

Figure S3. The principal components analysis (PCA) of common species based on eight seed traits (The PCA loadings for each seed trait were provided in Table S2; SN: seed number, SS: seed size, SL: seed length, SW: seed width, SH: seed height, SCC: seed carbon content, SNC: seed nitrogen content, SPC: seed phosphorus content).

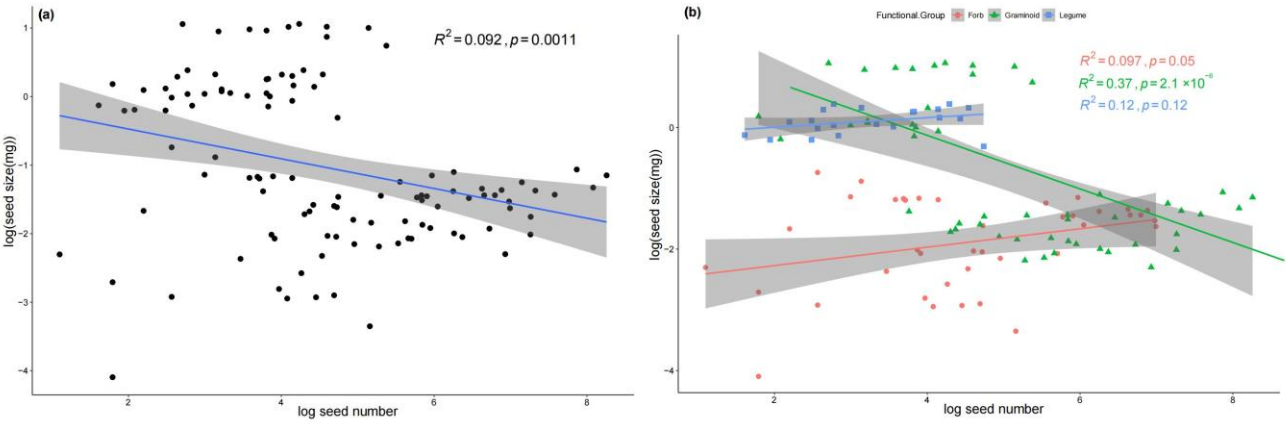

Figure S4. Relationships between seed size and seed number at the community level (**a**,**c**) and at the functional group level (**b**,**d**) after two years data pooled. The seed size and seed number data were log transformed.
